# Adopting wearables to customize health insurance contributions: a ranking-type Delphi

**DOI:** 10.1186/s12911-022-01851-4

**Published:** 2022-04-27

**Authors:** Daniel Neumann, Victor Tiberius, Florin Biendarra

**Affiliations:** 1 University of Potsdam, Potsdam, Germany; 2 Avanos Medical, Hamburg, Germany

**Keywords:** Delphi study, Health insurance, Wearable electronic device, Wearable technology, Internet of Things, Barriers

## Abstract

**Background:**

Wearables, as small portable computer systems worn on the body, can track user fitness and health data, which can be used to customize health insurance contributions individually. In particular, insured individuals with a healthy lifestyle can receive a reduction of their contributions to be paid. However, this potential is hardly used in practice.

**Objective:**

This study aims to identify which barrier factors impede the usage of wearables for assessing individual risk scores for health insurances, despite its technological feasibility, and to rank these barriers according to their relevance.

**Methods:**

To reach these goals, we conduct a ranking-type Delphi study with the following three stages. First, we collected possible barrier factors from a panel of 16 experts and consolidated them to a list of 11 barrier categories. Second, the panel was asked to rank them regarding their relevance. Third, to enhance the panel consensus, the ranking was revealed to the experts, who were then asked to re-rank the barriers.

**Results:**

The results suggest that regulation is the most important barrier. Other relevant barriers are false or inaccurate measurements and application errors caused by the users. Additionally, insurers could lack the required technological competence to use the wearable data appropriately.

**Conclusion:**

A wider use of wearables and health apps could be achieved through regulatory modifications, especially regarding privacy issues. Even after assuring stricter regulations, users’ privacy concerns could partly remain, if the data exchange between wearables manufacturers, health app providers, and health insurers does not become more transparent.

## Introduction

The basic idea of health insurance is to collect contributions from insured individuals and to cover their medical expenses, based on the principle of solidarity [1]. When individual health risks differ significantly, insurance contributions can be customized based on individual risk assessment and a healthy lifestyle. Individuals with higher health risks, such as pre-existing conditions or unhealthy behaviors, pay a premium, while participants with healthy behavior are rewarded with contribution reductions.

Cost-effective healthcare solutions using (smart) wearables have the potential to lower the insurance contribution for individual users by fostering healthy behavior and offering the possibility to collect health-related data used to assess individual health risks [2]. As part of the Internet of Things (IoT) [3–7], wearables are small portable computer systems, such as smart watches, that are worn on the body or clothing [8–10] and that monitor various health measures using biosensors [11]. They have become increasingly popular in recent years [12–14] and are developing into an integral part of personal analytics, measuring physical condition, recording physiological parameters or information about medication plans [15]. Combined with health apps, wearables are considered beneficial in areas such as increased fitness, preventive healthcare (prophylaxis) and chronic disease monitoring [16, 17], which leads to both health benefits and cost savings [18–20]. Furthermore, telemedicine services through wearable-based health monitoring can lead to improved access to healthcare for an ageing society like Germany by reducing in-person clinic appointments [3, 4, 21]. Additionally, data could be tracked continuously rather than with long breaks. Therefore, wearables and health apps can be cost-effective digital solutions for the wider sector of the health system [22, 23], representing a radical innovation [24] partly disrupting traditional health monitoring. The recent COVID-19 pandemic, with personal contact restrictions, has particularly shown the benefits of such telemedicine approaches. Patients can be informed about their health status by voice-controlled intelligent assistants (VIPAs) [25] or by doctors using social media channels [26], based on data from their wearables.

Despite these advantages, the actual usage of wearables for assessing individual risk scores for health insurance currently remains limited. The objective of this study is to identify and rank the barrier factors influencing wearable usage for assessing individual risk scores for health insurances in Germany. The conclusions from this study may be applicable for other countries as well.

Some previous research provides hints for several barriers of using wearables for insurance contribution customization. The acceptance of wearables and the collection of individual health data is key to their effectiveness [4]. A significant share of patients may dislike or refuse to be monitored by third parties. In a survey, it was found that 33% of respondents from 16 different countries used wearables and health apps to record their fitness or health [27]. Considering the novelty of this technology, this share is already relatively high. However, the majority still lacks interest or may find the technology too expensive [28]. Among the users of wearables, only 1% used devices sponsored by their health insurers [29]. This suggests that there is a high customer acceptance to use wearables and to track health data for personal use, whereas sharing this data with health insurers is less accepted.

The reason for this restraint might be data security and privacy concerns [28]. Such concerns are not unfounded as the majority of free health apps, according to their data protection guidelines or general terms, share the data with third parties [30–32]. The users’ major concern is that, due to the lack of privacy, their shared health data may lead to discrimination because of possible illnesses or a lifestyle considered unhealthy [33–36]. These concerns could only be reduced by assuring stricter state regulations for health data processing using health apps [6]. Another problem could be the users’ lack of knowledge when interpreting the collected health data correctly [37]. Additionally, wrong or imprecise collected health data due to the low quality of the devices’ sensors could lead to falsely calculated customized insurance contributions [37].

Apart from barriers on the users’ side, also barriers on the healthcare providers’ side might challenge the use of wearables to customize health insurance contributions, such as the integration of wearable-tracked health data into the healthcare organization, micropolitics, and missing incentives [38].

The aforementioned research focuses on single barrier factors. To the best of our knowledge, no study with a holistic view on barrier factors exists, and none used a ranking-type Delphi study design. With this study, we contribute to both research on fair health insurance contributions and research on wearables by exploring barrier factors relating to their use to customize insurance contributions. The findings can help insurers, wearable manufacturers, and health app developers to avoid shortcomings that impede customized health insurance contributions and adverse properties of wearables and health apps. The results of the study imply that regulation is the most pressing barrier factor. Another major problem are false or inaccurate measurements and application errors caused by the users.

The remainder of the paper is structured as follows: First, we explain the Delphi study design as the applied methodology of our research. Second, we present the results of the empirical study. Third, we discuss the findings, their implications, and the limitations of the study. Fourth, in the conclusion, we sum up the gained insights.

## Methodology

To achieve our research goal, we employ a ranking-type Delphi study. Delphi studies are a widely accepted and well-established research method [39–41]. Delphi studies are expert group surveys, which are considered to be more accurate and valid than both single expert interviews and layperson group surveys [42, 43]. Cognitive biases are reduced and allow for higher objectivity [43–45]. Among the different types of Delphi methods, we chose the ranking-type, which allows for the identification and ranking of influencing factors [41, 46], such as barriers.

The Delphi study consists of three stages: collecting and consolidating the barriers, ranking them, and re-ranking them [40, 46]. In the first phase, the brainstorming phase, the experts were asked to list possible barriers to the use of wearables to customize health insurance contributions. The experts were asked to indicate as many perceived barriers as possible. The minimum requirement was to specify three possible barriers. A total of 58 barriers were mentioned by the experts. Consolidating the list of barriers was carried out in three steps. First, each statement was copied onto a table. Second, the statements that had the same meaning were then grouped to barrier categories. Third, duplicates and statements belonging to the same category were removed. As a result, a total of eleven barrier categories remained. Narrowing down the list was unnecessary due to the manageable number of entries [47].

In the second stage, the experts were asked to arrange the randomly sorted barriers according to their relevance, with first place being the most relevant. The third stage, the re-ranking stage, aimed to increase the group consensus, as a constitutive characteristic of the Delphi study design, by giving structured feedback about the results from the second round [43, 46, 48–52]. Therefore, the ranked list from the second round was provided to the panel, and the experts were asked to make adjustments if necessary. The survey was conducted anonymously.

Delphi studies use a purposive sampling method [53], based on the respondents’ thematic expertise [41, 43, 45, 54–57]. The number of respondents is usually rather small [40, 55], with seven considered as the minimum [48] and usually comprising 15 to 35 experts [54]. To select our sample, we searched for professionals working with health insurance organizations and manufacturers of wearables. For our study, it would potentially also have been reasonable to include wearable users as survey participants. However, we followed the conventional Delphi study protocol, which prescribes to exclude laypersons [42, 43]. We address this further in the limitations.

Potential experts were contacted by telephone. A total of 20 participants agreed to take part in the survey. Of them, 18 participated in the survey, marking a response rate of 90%. Two respondents did not fill in the survey completely. As a consequence, 16 surveys were used for the analysis. Between the rounds, the dropout rate was 0%. The first survey was carried out from November 2nd, 2020 to November 15th, 2020, the second survey from November 16th, 2020 to November 24th, 2020, And the third round started on November 25, 2020 and was completed on November 30th, 2020.

Table 1 depicts the panel profile. Seven experts were female (43.75%) and 9 were male (56.25%). The experts were 38.69 years old on average and had an average work experience of 9.72 years. Twelve respondents (75%) worked for health insurance companies and four (25%) worked for wearable manufacturers, all in the software department. The proportion of experts with a wearable background was deliberately kept low because we assumed that they may be subject to a desirability bias [44, 45], seeing more opportunities rather than barriers for wearables as working for a producer usually involves a high level of identification with the product [58].

**Table 1:** Panel description

Gender	Female	7 (43.75%)
	Male	9 (56.25%)
Age (years)	< 30	7 (43.75%)
	30–39	2 (12.50%)
	40–49	2 (12.50%)
	50–59	4 (25.00%)
	> 60	1 (6.25%)
	Mean	38.69
Work experience (years)	< 6	7 (43.75%)
	6–10	4 (25.00%)
	> 10	5 (31.25%)
	Mean	9.72
Occupation	Health insurer	12 (75.00%)
	Wearable manufacturer	4 (25.00%)

The ranking and the re-ranking were analyzed as follows: The average ranking place for each barrier *b* was calculated as the arithmetic mean ${\overline{r} }_{b}$ of the ranks of this barrier provided by all respondents (N = 16):

$${\overline{r} }_{b}=\frac{1}{N}\sum_{i=1}^{N}{r}_{b,i}.$$

We also calculated the standard deviation σ as a measure of the panel disagreement:

$$\sigma = \sqrt {\frac{1}{N - 1}} \mathop \sum \limits_{i = 1}^{N} (r_{b,i} - \overline{r}_{b} )^{2} .$$

The Delphi study was conducted in accordance to the voluntary “Research guidelines for the Delphi survey technique” [55]. As the survey did not include any medical or clinical experimentation or ethically relevant human research, the ethics commission of our university was not competent for this kind of research and an approval was neither needed nor possible.

## Results

The results from the ranking and re-ranking are depicted in Table 2. The final results from the third-round show that regulation barriers (i.e., privacy issues and data protection as well as other legal requirements) are the most important ones. In the third and fourth places follow inaccurate measurements and application errors as issues the users are responsible for. The fifth most relevant barrier addresses the insurers, particularly their lack of technological competence to use the wearable data appropriately. Barriers six and seven are principal considerations, namely that data from wearables is generally not suitable to calculate individual contributions and that a contributions customization is not compatible with the solidary principle of insurances. Less relevant barriers are a lack of people’s acceptance of wearing wearables in public, a bad cost–benefit ratio of individual contributions, a lack of access to wearables, and fear of higher insurance contributions as a consequence of suggested unhealthy behavior.

**Table 2:** Ranked barriers in the second and third survey round

Second round				Third round			
Place	Barrier	$\overline{r }$	σ	Place	Barrier	$\overline{r }$	σ
1	Privacy issues/data protection	2.88	2.34	1	Privacy issues/data protection	1.50	0.87
2	Legal requirements	3.81	2.94	2	Legal requirements	2.94	2.08
3	Lack of data validity/inaccurate measurements	4.38	3.06	3	Lack of data validity/inaccurate measurements	4.63	2.37
4	Application errors	5.25	2.08	4	Application errors	4.69	2.05
4	Solidarity principle of insurance	5.25	3.54	5	*Lack of technological competencies at health insurers*	6.13	2.57
5	Lack of technological competencies at health insurers	6.50	2.81	6	*Wearable data not suitable for contribution adjustments*	6.25	2.14
6	Wearable data not suitable for contribution adjustments	7.00	2.94	7	*Solidarity principle of insurance*	6.63	2.03
7	Lack of public acceptance of wearing wearables	7.56	2.45	8	Lack of public acceptance of wearing wearables	7.00	2.62
8	Bad cost–benefit ratio of individual contributions	7.63	2.47	9	Bad cost–benefit ratio of individual contributions	8.38	2.74
9	Lack of access to wearables	7.88	2.29	10	Lack of access to wearables	8.69	2.05
9	Fear of sanctions	7.88	2.03	11	Fear of sanctions	9.19	1.88

$\overline{r }$: average rank; σ: standard deviation. Shifts in italics

Whereas in the second round, several barriers were seen as equally relevant, the third round led to clearer differentiations. The order of the barriers hardly changed. Only the three barriers on places five to seven, i.e., the medium-relevant barriers, saw slight shifts. A possible lack of technological competence at health insurers and the missing suitability of data from wearables for contributions adjustments were seen as slightly more relevant in the third round, whereas the violation of the solidarity principle was rated as less relevant. These only slight changes suggested that a fourth survey round was not necessary. This is further suggested by an increased consensus in the group, which can be measured by a reduction of the average standard deviation. The average standard deviation was 2.63 in the second round, whereas it was 2.13 in the third round.

## Discussion

### Interpretation of the results and future research

In both surveys, the two most important barriers (privacy/data protection and other legal requirements) address regulatory issues. These issues correspond with the findings in previous research [6, 7, 28, 34, 59, 60]. As both the health care sector and the health insurance sector are highly regulated, it is not surprising that the respondents pay special attention to such issues. However, these barriers are the responsibility of neither health insurers nor users and cannot be overcome by them directly. Health insurers could try to exert influence by lobbying. Similarly, users could start petitions. If states handle the regulatory issues in a manner that creates legal certainty and is considered satisfactory by health insurers and especially by users, the main barriers could be overcome.

Inaccurate measurements and application errors are within the users’ responsibility and are also mentioned by previous research [37]. These issues are particularly important for health insurers, as data generated by wearables must have a minimum level of reliability and have to be free from manipulation. If this is not the case, there is a risk that contributors manipulate their transmitted data to their advantage and thereby reduce the contributions to be paid, although the actual health data would not allow this. However, it is not clear if these barriers really exist or are just the insurers’ concerns. Empirical observations could clarify this. We call on future research to investigate this. If the assumption is confirmed, periodical or random measurements in medical practices could be executed. Handling errors, without the intention to manipulate data, could be diminished by (online) trainings and tutorials.

It is unclear if health insurers really show a lack of technological competence to use wearable data. This also should be found out by future studies. However, it is questionable if insurers really need that competence or, more likely, IT service providers will be used to handle data management. In either case, it appears to be less of a problem to overcome the potential lack of technological competence.

Overall, other less relevant barriers, such as a lack of acceptance for wearing wearables in public [4, 28] or the potential fear of sanctions [33–36], confirm considerations in previous research, whereas new barriers could be identified and rated.

As a consequence of the results of the present study, it can be stated that a legally certain framework for the use of data generated by wearables is the major barrier for the individual adjustment of health insurance contributions [7, 29, 59, 60]. A wider use of wearables and health apps could be achieved if some regulatory modifications were implemented. They could create transparency and trust. However, even if data protection laws become stricter, the users’ privacy concerns could partly remain. More transparency regarding the collaboration between wearables manufacturers, health app providers, and health insurers could lead to an increased trust among users. For example, health insurers could test and certify both wearable devices and health apps or develop their own health apps to ensure that the sensitive generated health data is only forwarded to the health insurer and its legitimate IT service providers but no other parties. Apart from the privacy issues, barriers within the users’ and health insurers’ responsibility appear comparably easy to overcome.

Beyond a strict focus on barrier factors impeding the use of wearables for the customization of insurance contributions, we encourage future research to examine other potential benefits of wearables. In particular, for which specific disease states can wearables be used to improve the patients’ situation? For example, current research exists on chronic obstructive pulmonary disease (COPD) [61], depression [62], distress [63], multiple sclerosis [64], Parkinson's disease [65], post-surgery complications [66], and sleep apnea [67]. We believe that many further application fields exist.

### Limitations

Our study comes with several limitations. First, even if the sample size meets the requirements of Delphi studies, future studies could consider larger or differently structured panels. In particular, the number of wearables manufacturers could be increased, and developers of health apps could be included. As mentioned before, this could potentially increase the risk of a desirability bias [44, 45], on the one side. On the other side, wearables manufacturers and health app developers could also see further barrier factors in the first survey round. Additionally, even if this leads to a higher standard deviation, this would represent an interesting insight.

Also insured individuals as (potential) users of wearables could be included. On the one side, this would violate the requirement of Delphi studies, which only allows the participation of experts. On the other side, as the most relevant stakeholder group, insured individuals could possibly identify further barrier factors wearables manufacturers and health app developers do not have in mind.

Second, interpreting the sorted list of obstacles, it has to be kept in mind that these are only ranked in order of their relevance, whereas the exact distances between the barriers remain unclear. For example, the first barrier could impede the use of wearables for the adaption of health insurance contributions ten times as much as the second barrier and 25 times as much as the third barrier. A ranking-type Delphi cannot provide such insights. Other survey formats could expand on our findings.

## Conclusion

In the present study, the barriers to the use of wearables and health apps for the adjustment of health insurance contributions were identified and ranked. For this purpose, a three-stage ranking-type Delphi study with 16 experts was carried out. The results suggest a total of eleven barrier categories, with a focus on legal and technological barriers. A regulatory framework for the use of wearables for health insurance matters has to be ensured before barriers within the direct responsibility of users, health insurers, wearables manufacturers, and health app providers could be overcome.

## Data Availability

All data generated or analyzed during this study are included in this published article.
